# TLR2 regulates angiotensin II-induced vascular remodeling and EndMT through NF-κB signaling

**DOI:** 10.18632/aging.202290

**Published:** 2020-12-09

**Authors:** Ke Lin, Wu Luo, Jueqian Yan, Siyuan Shen, Qirui Shen, Jun Wang, Xinfu Guan, Gaojun Wu, Weijian Huang, Guang Liang

**Affiliations:** 1 Chemical Biology Research Center, School of Pharmaceutical Sciences, Wenzhou Medical University, Wenzhou 325035, Zhejiang, China; 2 Department of Cardiology, The First Affiliated Hospital of Wenzhou Medical University, Wenzhou 325035, Zhejiang, China; 3 Department of Cardiology, Wenzhou Central Hospital and Affiliated Dingli Clinical Institute, Wenzhou Medical University, Wenzhou 325035, Zhejiang, China; 4 Affiliated Cangnan Hospital, Wenzhou Medical University, Cangnan 325800, Zhejiang, China

**Keywords:** endothelial to mesenchymal transition, TLR2, angiotensin II, vascular remodeling, NF-κB

## Abstract

Excessive vascular remodeling has been shown in hypertensive patients. In experimental models of hypertensive vascular injury, such as angiotensin II (Ang II) challenged mice, toll like receptor 2 (TLR2) initiates inflammatory responses. More recently, studies have reported atypical endothelial to mesenchymal transition (EndMT) in vascular injuries and inflammatory conditions. Here, we aimed to investigate whether TLR2 mediates Ang II-induced vascular inflammation and initiates EndMT. In a mouse model of angiotensin II-induced hypertension, we show that aortas exhibit increased medial thickening, fibrosis, and features of EndMT. These alterations were not observed in TLR2 knockout mice in response to Ang II. TLR2 silencing in cultured endothelial cells confirmed the essential role of TLR2 in Ang II-induced inflammatory factor induction, and EndMT-associated phenotypic change. Mechanistically, we found Ang II activates nuclear factor-κB signaling, inducing pro-inflammatory cytokine production, and mediates EndMT in both cultured endothelial cells and in mice. These studies illustrate a novel role of TLR2 in regulating Ang II-induced deleterious vascular remodeling through the induction of EndMT. The studies also suggest that TLR2 may be targeted to alleviate hypertension-associated vascular injury.

## INTRODUCTION

Hypertension is an important risk factor of adverse cardiovascular and cerebrovascular events, affecting more than 1 billion people worldwide [1, 2]. Hypertension-associated morphologic and functional changes include alterations in the vasculature and the development of endothelial dysfunction. In response to persistently elevated blood pressure and an imbalance of circulating cytokines, vascular tissues undergo progressive changes of increased extracellular matrix deposition, hyperplasia of smooth muscle cells, and altered vessel resistance [3]. Although the mechanisms are not fully clear, recent studies have shown that angiotensin II (Ang II) is capable of mediating these changes [4, 5]. Studies that elucidate the mechanisms underlying these Ang II activities, whether hemodynamic or nonhemodynamic, are valuable to identify targets for therapy.

Vascular endothelial cells can acquire a mesenchymal phenotype upon irritation or injury, in a process termed endothelial-to-mesenchymal transition (EndMT) [6]. During this cell phenotype switch, endothelial cells undergo a morphological change from a flatted to a fusiform shape, lose their endothelial-selective markers such as CD31 and vascular endothelial-cadherin (VE-cadherin), and begin to express genes associated with mesenchymal cells such as vimentin and α-smooth muscle actin (α-SMA). As this change progresses, cells become increasingly migratory, and contribute to expansive vascular remodeling [7]. EndMT can be induced by a number of factors. One such factor is transforming growth factor-β1 (TGF-β1). Activation of the TGF-β/Smad signaling pathway induces EndMT transcription factors Snai1 and Snai2 (also known as Slug) that promote EndMT-related changes in cells [8]. It is possible that Ang II also induces EndMT during hypertension-associated vascular remodeling and injury. Xu and colleagues demonstrated that endothelial-specific deletion of Ets-1 attenuates Ang II-induced cardiac fibrosis by suppressing EndMT [9]. In another study, Liu et al showed that a bioactive compound kaempferol suppresses Ang II-induced EndMT in heart tissues and reduces cardiac fibrosis [10].

Although most of the actions of Ang II are attributed to angiotensin II type 1 receptor, recent studies have identified toll like receptors (TLRs) in nonhemodynamic actions of Ang II, especially in the context of disease [11–13]. For example, TLR2 has been shown to regulate Ang II-induced inflammatory responses in heart and kidney tissues [14, 15], and during aortic aneurysms [16] and aortic stenosis [17]. Considering the fact that inflammation is also an inducer of EndMT and endothelial dysfunction [18], we hypothesized that Ang II engages TLR2 to induce EndMT and result in deleterious vascular remodeling. To test this hypothesis, we utilized TLR2 knockout mice and generated vascular dysfunction by exogenous Ang II administration. We show that Ang II activates EndMT-associated changes in aortas of mice, manifesting as induction of EndMT-regulating transcription factors, suppressed levels of endothelial markers, and increased expression of mesenchymal markers. We found that these changes are associated with increased inflammatory responses and matrix protein deposition. TLR2 knockout mice are completely protected against these pathological changes induced by Ang II. We also found that nuclear factor-κB is intricately involved as its inhibition mimics TLR2 deficiency. These results highlight the novel engagement of TLR2 by Ang II to induce EndMT and vascular alterations, and identify TLR2 as a potential therapeutic target.

## RESULTS

### TLR2 knockout mice are protected against Ang II-induced fibrosis in aortas

**Figure 1:**
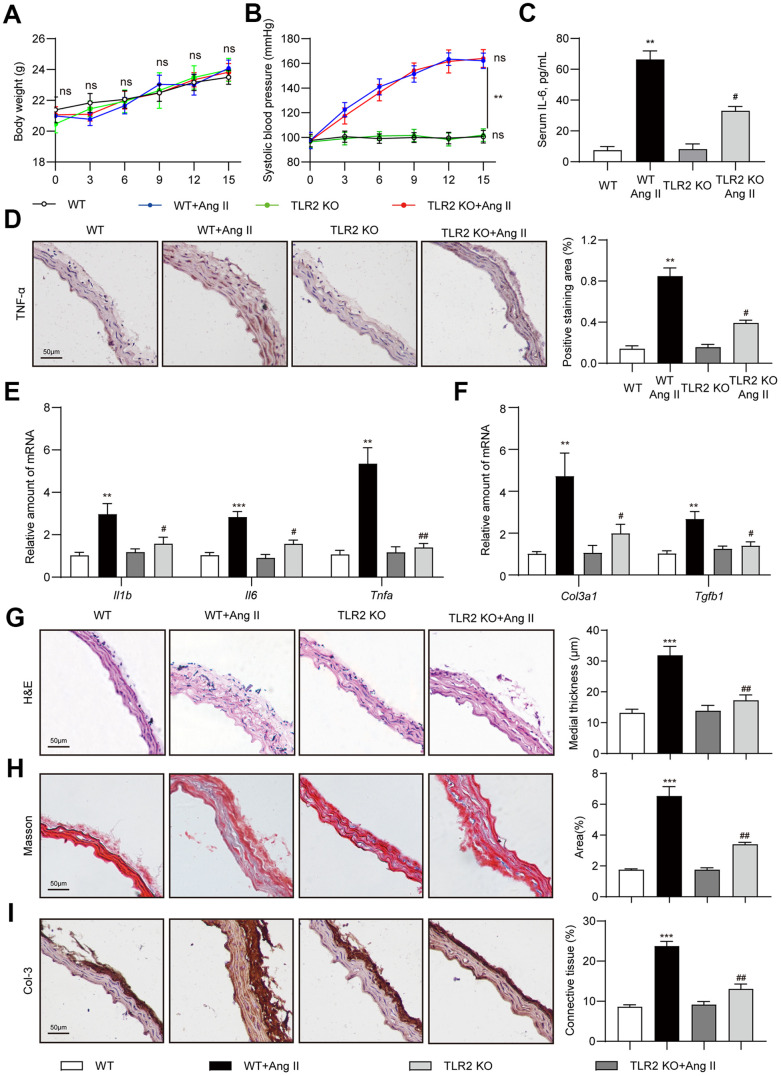
**TLR2 deficiency prevents Ang II-induced vascular remodeling in mice.** Wildtype (WT) and TLR2 knockout (TLR2KO) mice were challenged with Ang II for 2 weeks. Data showing (**A**) body weights of mice, (**B**) systolic blood pressure measurements, and (**C**) levels of serum interleukin-6 (IL-6). (**D**) Representative images of TNF-α staining of mouse aortic tissues [scale bar = 50 μm]. Quantification of TNF-α staining area is shown on right. (**E**, **F**) mRNA levels of pro-inflammatory and fibrosis-associated genes in aortic tissues [data normalized to β-actin]. (**G**, **H**) H&E and Masson's Trichrome staining of mouse aortic sections illustrating the degree of medial thickening and fibrosis [scale bar = 50 μm]. Quantification of medial thickening and connective tissue deposition are shown on right. (**I**) Representative images of Col-3 staining (brown) of aortic sections. Tissues were counterstained with hematoxylin (blue) [scale bar = 50 μm]. Quantification of Col-3 staining is shown on right. [n = 6-8; Data shown as Mean ± SEM; **p<0.01, and ***p<0.001 compared to WT; #p<0.05 and ##p<0.01 compared to WT-Ang II].

In order to investigate the role of TLR2 in Ang II-induced deleterious vascular remodeling, we challenged mice with low-dose Ang II infusion for 2 weeks. We did not observe difference in body weights of mice following Ang II infusion (Figure 1A). TLR2 knockout (TLR2 KO) mice also did not exhibit body weight differences compared to wildtype (WT) mice. Ang II infusion elevated systolic blood pressure in both WT and TLR2 KO mice as expected (Figure 1B). Since chronic inflammation contributes to vascular remodeling [19], and TLR2 regulates inflammatory responses [20], we measured the levels of serum interleukin-6 (IL-6) as a proxy for inflammatory status. Our results show significantly elevated serum IL-6 in WT mice challenged with Ang II (Figure 1C). The level of IL-6 induction was significantly dampened in TLR2 KO mice. Consistent with these findings, TNF-α immunoreactivity was increased in aortas of WT mice upon Ang II exposure but not in aortas from TLR2 KO mice (Figure 1D). We confirmed these findings by measuring mRNA abundance of pro-inflammatory genes and show that TLR2 KO mice are protected against Ang II-induced TNF-α, IL-1β and IL-6 (Figure 1E). This inflammatory readout mimicked expression patterns of fibrosis-associated genes transforming growth factor-β1 (TGF-β1) and collagen type 3 (col-3) (Figure 1F). Importantly, the results show that Ang II causes increased levels of proinflammatory and fibrotic factors in wildtype mice, and that TLR2 knockout mice show significantly dampened responses to Ang II. We then examined aortic tissues of mice and show that Ang II increases medial thickness in WT mice but not TLR2 KO mice (Figure 1G). Trichome staining and collagen 3 immunoreactivity confirmed increased deposition of connective tissue in aortas of wildtype mice (Figure 1H, 1I). Consistent with other analyses, TLR2 KO mice did not exhibit increased fibrosis in response to Ang II challenge.

### Deficiency of TLR2 prevents Ang II-induced EndMT in aortas

**Figure 2:**
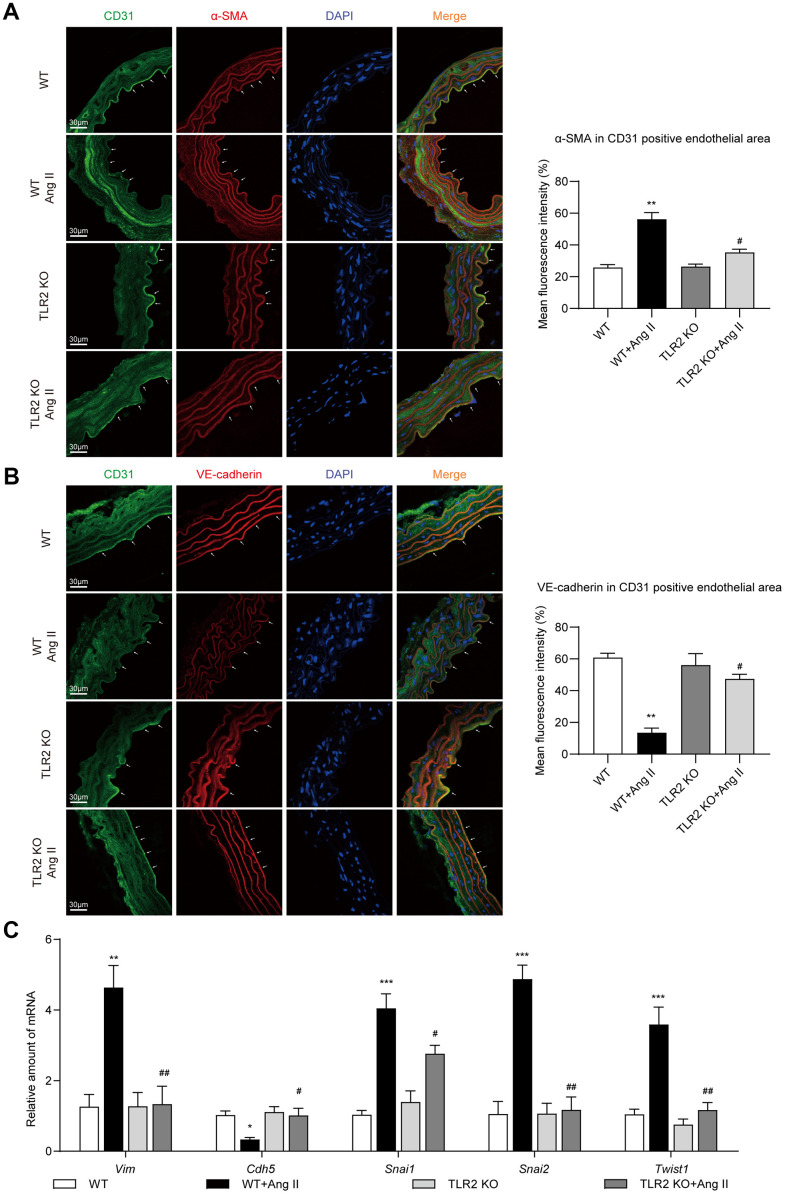
**TLR2 KO mice are protected against Ang II-induced EndMT in aortas.** (**A**) Representative immunofluorescence staining images showing CD31 (green) and α-SMA (red) in mouse aortic tissues. Arrows indicate positive staining. Tissues were counterstained with DAPI (blue) [scale bar = 30 μm]. Quantification of α-SMA fluorescence in CD31-positive endothelial area is shown on right. (**B**) Representative immunofluorescence staining images showing CD31 (green) and VE-cadherin (red) in mouse aortic tissues. Arrows indicate positive staining. Tissues were counterstained with DAPI (blue) [scale bar = 30 μm]. Quantification of VE-cadherin in CD31-positive endothelial area is shown on right. (**C**) mRNA levels of EndMT-associated genes in aortas [Data normalized to β-actin]. [n = 6-8; Data shown as Mean ± SEM; *p<0.05, **p<0.01, and ***p<0.001 compared to WT; #p<0.05 and ##p<0.01 compared to WT-Ang II].

Accumulating evidence suggests that EndMT bridges, or at least is associated with, inflammation and vascular injury [18]. To determine whether regulation of EndMT was involved in the protective phenotype of TLR2 KO mice against Ang II, we first performed immunofluorescence labeling of aortic tissues. We stained the sections for endothelial marker CD31 and mesenchymal marker α-SMA. A decrease in CD31 and an increase in α-SMA immunoreactivity was seen in tissues of wildtype mice upon Ang II challenge (Figure 2A). TLR2 KO mice, however, showed significantly less alterations as compared to wildtype mice. Similar to CD31 staining, we also observed levels of vascular endothelial-cadherin (VE-cadherin) to be suppressed by Ang II in WT mice (Figure 2B). No such decreases were noted in TLR2 KO mice challenged with Ang II. Next, we measured mRNA levels of EndMT-associated genes in aortic tissues and found increased levels of *Twist1*, *Snai1*, *Snai2* (slug) and *Vim* (vimentin) in WT mice after Ang II exposure (Figure 1C). In addition, *Cdh5* (VE-cadherin) mRNA levels were reduced upon Ang II administration. These data suggest an increase in mesenchymal cell transcripts in aortas of mice following Ang II infusion. These mRNA alterations were largely absent in TLR2 KO mice. Collectively, these results indicate that TLR2 may be involved in Ang II-induced EndMT in vascular tissues.

### TLR2 silencing prevents EndMT-associated changes in cultured endothelial cells

We performed our *in vivo* studies using whole tissue lysates, which makes it difficult to pinpoint the source of these mRNA and protein alterations. Although we focused on the endothelial lining in our immunofluorescence studies, we need to confirm the role of TLR2 in regulating Ang II-induced EndMT in purified endothelial cell system. To do this, we utilized cultured HUVECs and exposed the cells to Ang II. Initially, we performed an Ang II dose-response and determined how rapidly EndMT-associated proteins are altered. Our results show that 36 h is enough to significantly induce EndMT transcription factors Twist1 and Snail/Slug (Supplementary Figure 1A, 1C). These transcription factors are induced in parallel to increases in mesenchymal markers α-SMA and vimentin, increases in collagen 3, and decreases in endothelial cadherin (Supplementary Figure 1A, 1C). Dose-response studies pointed to 10 μM and higher levels of Ang II mediating EndMT in HUVECs (Supplementary Figure 1B, 1D). Based on these results, we used 10 μM Ang II for subsequent studies. Although higher concentrations of Ang II also induced EndMT in a robust manner, we have elected to use the lowest concentration that elicits a response, in an attempt to avoid saturating and potentially confounding conditions expected from higher concentrations.

**Figure 3:**
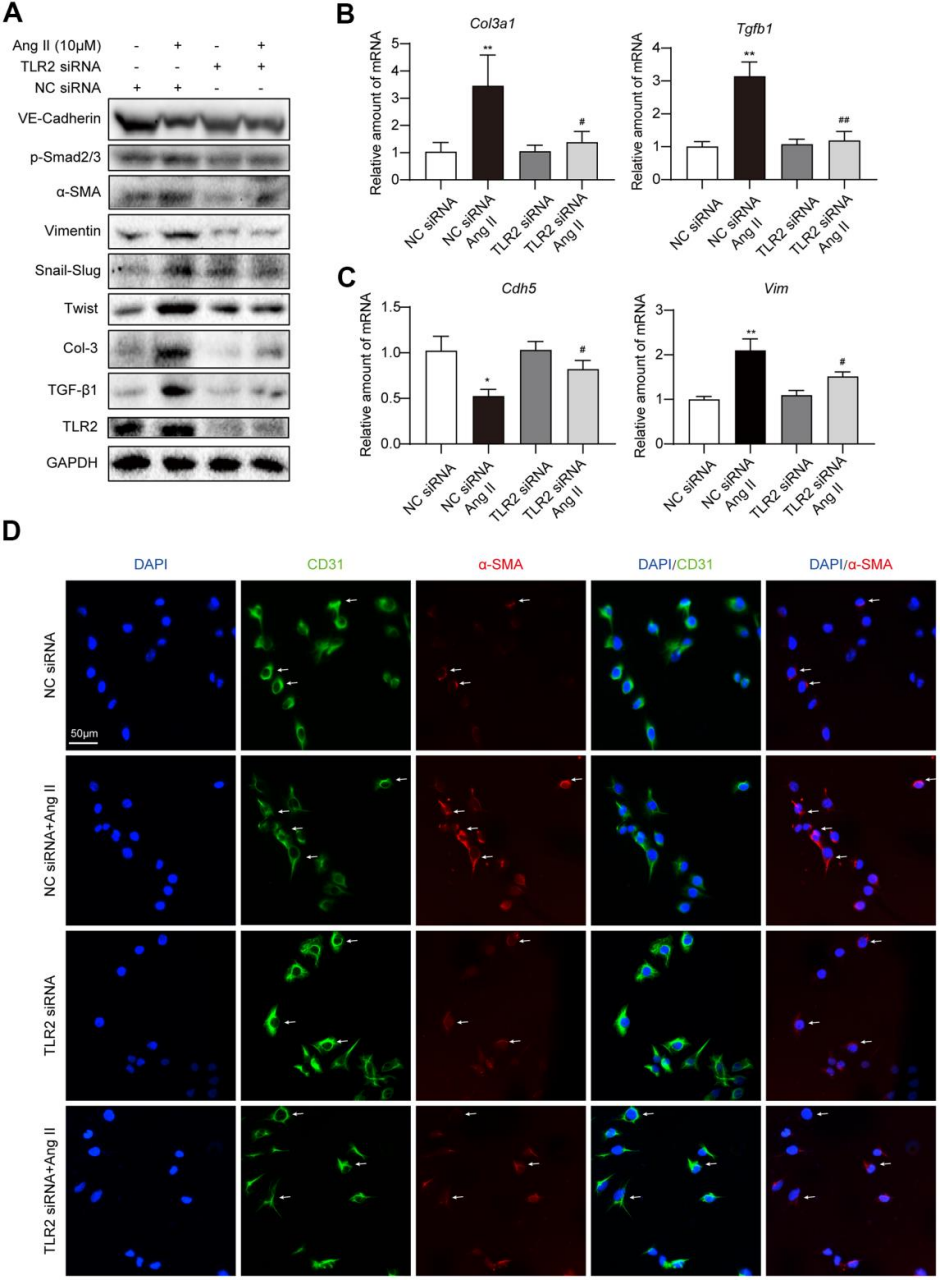
**Silencing TLR2 prevents EndMT phenotype in cultured endothelial cells.** (**A**) HUVECs were transfected with siRNA against TLR2. Control cells were transfected with negative control (NC) siRNA. Cells were then challenged with 10 μM Ang II for 36 h. Total proteins were probed for TLR2 and EndMT-associated proteins. GAPDH was used as loading control. (**B**, **C**) HUVECs were exposed to 10 μM Ang II for 24 h, after siRNA transfections. mRNA levels of EndMT-associated genes were determined. Data was normalized to β-actin. (**D**) Representative immunofluorescence staining of HUVECs for CD31 (green) and α-SMA (red). Cells were transfected with indicated siRNA and then exposed to 10 μM Ang II for 36 h. Slides were counterstained with DAPI (blue). Arrows indicate HUVECs undergoing EndMT, as evident through loss of CD31 and induction of α-SMA [Scale bar = 50 μm]. [n = 3; Data shown as Mean ± SEM; *p<0.05 and **p<0.01, compared to negative control transfection; #p<0.05 and ##p<0.01 compared to Ang II challenged negative control transfection].

To assess a potential role of TLR2 in EndMT, we knocked down the levels of *TLR2* by siRNA transfection (Supplementary Figure 2) prior to Ang II challenge. Our results indicate that *TLR2* deficiency prevents EndMT induction by Ang II (Figure 3A, Supplementary Figure 3A–3I). Specifically, an increase in Snail/Slug, Twist1, and α-SMA and vimentin were not observed in HUVECs exposed to Ang II following *TLR2* siRNA transfection. We also did not observe alterations in VE-cadherin and fibrosis-associated TGF-β1 and Col-3 in cells upon TLR2 silencing. To supplement these findings, we probed for TGF-β1 signaling protein Smad2/3 and show that *TLR2* silencing prevents Ang II-induced levels of phosphorylated (activated) Smad2/3. mRNA levels of select genes from this panel confirmed that TLR2 potentially regulates Ang II-induced EndMT (Figure 3B, 3C, Supplementary Figure 3J). Immunofluorescence staining of cells exposed to Ang II confirmed the loss of CD31 and the induction of α-SMA in endothelial cells with TLR2 expression but not in cells in which *TLR2* was silenced (Figure 3D, Supplementary Figure 3K, 3L).

### TLR2 regulates EndMT through NF-κB signaling

**Figure 4:**
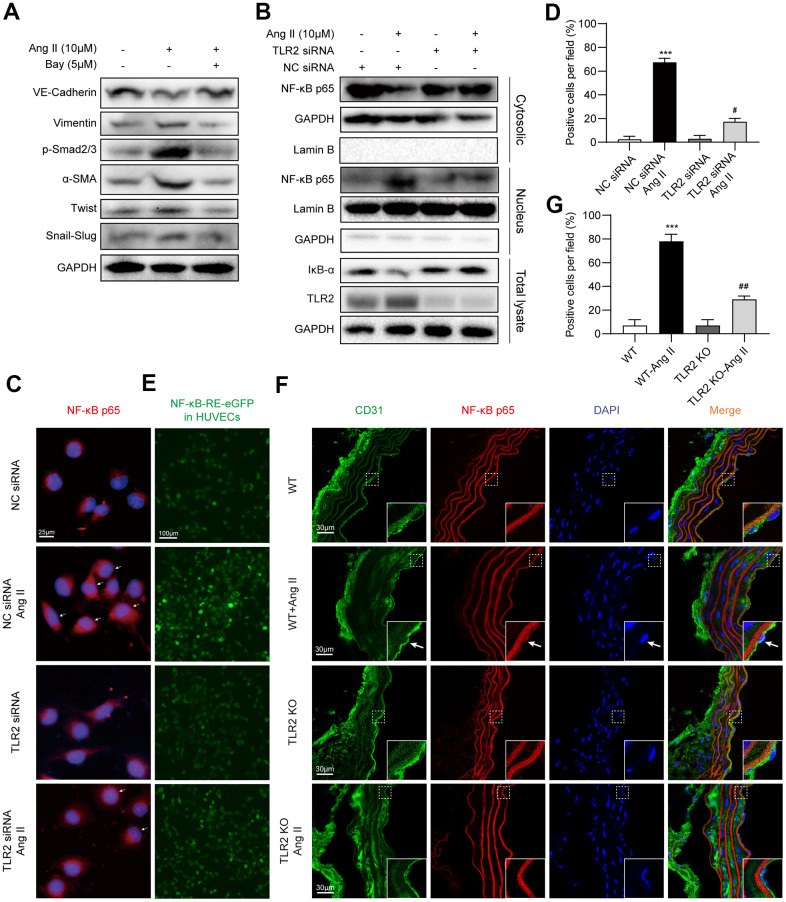
**TLR2 regulates EndMT through NF-κB.** (**A**) HUVECs were pretreated with NF-κB inhibitor Bay 11-7085 at 5 μM for 1 h. Cells were then exposed to 10 μM Ang II for 36 h. Levels of EndMT proteins were detected by immunoblotting. GAPDH was used as loading control. (**B**) HUVECs were transfected with TLR2 siRNA or negative control siRNA. Transfected cells were exposed to 10 μM Ang II for 2 h. Total proteins, and nuclear and cytoplasmic fractions were probed for NF-κB p65 subunit and inhibitor of κBα (IκB). GAPDH was used as loading control for total proteins and cytosolic fractions. Lamin B was used as loading control for nuclear fractions. (**C**) Cells treated as indicated in Panel B were stained for p65 subunit (red). Counterstaining with DAPI was performed [scale bar = 25 μm]. (**D**) Quantitative analysis of nuclear p65 staining in HUVECs. Representative staining images are shown in panel C. (**E**) HUVECs expressing NFκB-EGFP reporter were transfected with NC siRNA or TLR2 siRNA, and challenged with 10 μM Ang II for 2 h. Representative images of EGFP (green) fluorescence of each group are shown [scale bar = 100 μm]. (**F**) Immunofluorescence staining of aortic tissues for CD31 (green) and NF-κB p65 (red). Tissues were counterstained with DAPI [scale bar = 30 μm]. Inserts showing high-power images of tissues (arrows indicating endothelial cells). (**G**) Quantitative florescence intensity of p65 in tissues, expressed as percent positive cells. Representative fluorescence images are shown in panel F. [n = 3 in A-E; n = 7 per group in F and G; Data shown as Mean ± SEM; ***p<0.001 compared to negative control siRNA transfection (**D**) or wildtype mice (**G**); #p<0.05 compared to NC siRNA+Ang II (**D**) or WT-Ang II (**G**)].

Involvement of NF-κB signaling during EndMT has previously been documented [21], and may potentially be involved downstream of TLR2 activation in our experimental model. To assess this possibility, we pretreated HUVECs with a specific NF-κB inhibitor Bay 11-7805 and then exposed the cells to Ang II. We show that NF-κB inhibition, similar to *TLR2* silencing, prevents Ang II-induced EndMT phenotype switch in HUVECs (Figure 4A, Supplementary Figure 4). We next probed whether NF-κB is activated in HUVECs upon Ang II exposure. A decrease in cytosolic levels of p65 and an increase in nuclear p65 was noted in cells exposed to Ang II (Figure 4B, Supplementary Figure 5A–5D). This nuclear translocation of NF-κB was not seen in cells upon *TLR2* silencing. Degradation of the inhibitor of κBα (IκB) in response to Ang II was also observed to be prevented in cells following *TLR2* silencing. We confirmed the involvement of NF-κB activation in TLR2-mediated effects by staining cells. Results indicate increased nuclear p65 proteins upon Ang II challenge in control cells but not in *TLR2* siRNA-transfected cells (Figure 4C, 4D). Using the EGFP reporter for NF-κB transcriptional activity, we obtained similar results in NF-κB-EGFP-transfected HUVECs with scrambled siRNA or *TLR2* siRNA (Figure 4E, Supplementary Figure 5E). Specifically, *TLR2* silencing reduced Ang II-induced NF-κB activity. We also examined the levels of NF-κB p65 in aortic tissues of WT and TLR2 KO mice to confirm the involvement of NF-κB in this signaling axis. As expected, Ang II significantly increased aortic NF-κB p65 levels in WT mice, with a significantly suppressed response in TLR2 KO mice (Figure 4F, 4G).

### Ang II causes endothelial cells to release paracrine factors which mediate EndMT in a TLR2-independent mechanism

**Figure 5:**
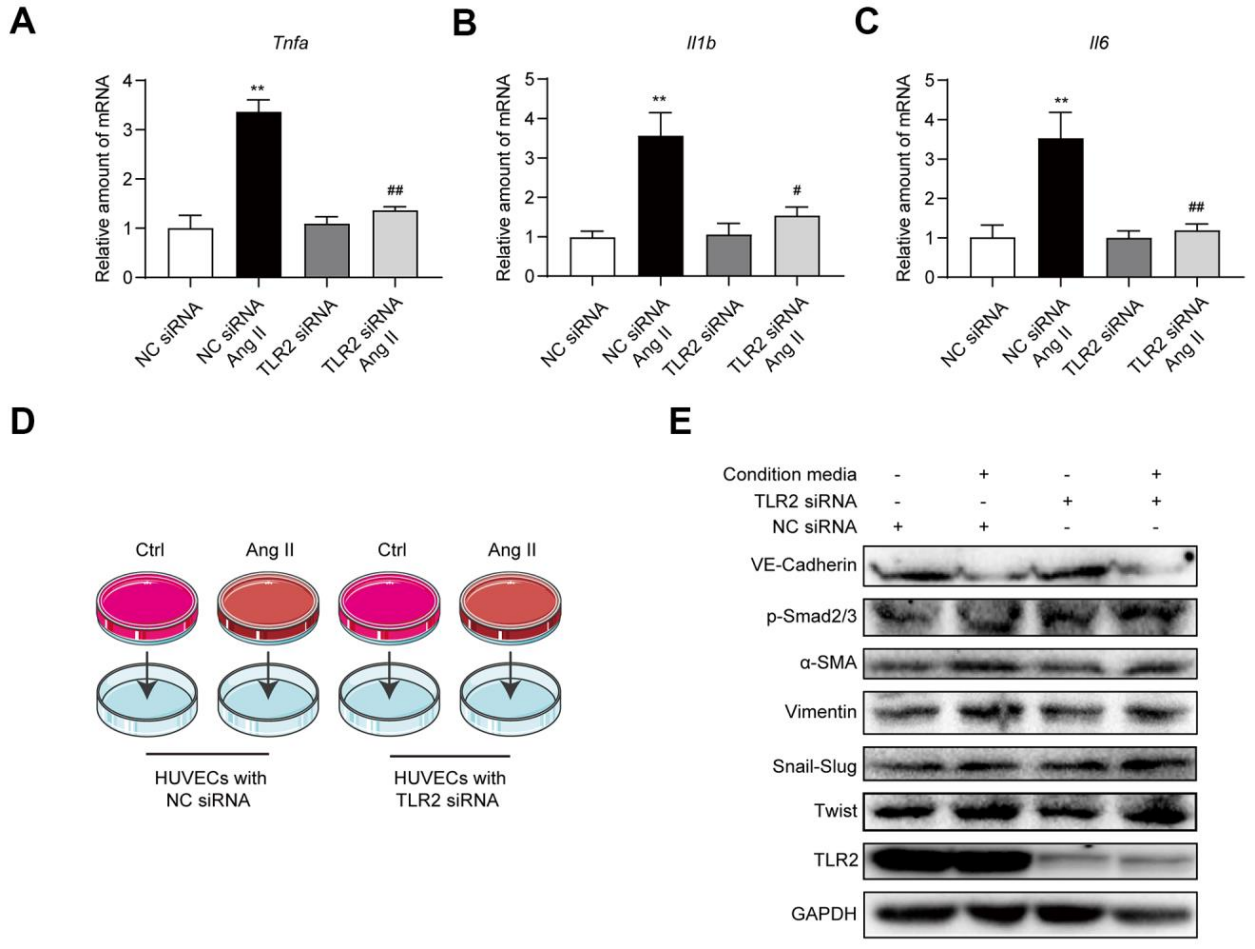
**Blockade of TLR2 signaling does not affect downstream processes of inflammatory factors and EndMT.** (**A**–**C**) HUVECs were transfected TLR2 or negative control siRNA. Cells were then exposed to 10 μM Ang II for 6h. mRNA levels of proinflammatory factors were detected [data normalized to β-actin]. (**D**) Schematic illustration of the experimental model to test the role of Ang II-induced paracrine factors in EndMT. Conditioned media was collected from cells challenged with 10 μM Ang II for 24 h. Media was then added to HUVECs transfected with TLR2 or negative control siRNA. (**E**) Levels of TLR2 and EndMT-associated proteins were determined in cells treated in panel D. GAPDH was used as loading control. [n = 3; Data shown as Mean ± SEM; **p<0.01 compared to negative control; #p<0.05 and ##p<0.01 compared to Ang II exposed negative control].

Our next objective was to determine whether Ang II-TLR2-NF-κB axis is involved in the elaboration of inflammatory factors. Exposure of HUVECs to Ang II showed higher levels of *Tnfa*, *Il6*, and *Il1b* mRNA levels (Figure 5A–5C). These inductions were prevented in HUVECs transfected with *TLR2* siRNA. Based on these data, we explored the possibility that Ang II-induced inflammatory and other paracrine factors may contribute to EndMT, in a pathway that may also depend on TLR2. To test this, we exposed HUVECs to Ang II and collected the condition media. This condition media, which comprised Ang II-induced inflammatory cytokines, was applied to a separate set of cells transfected with scrambled or *TLR2* targeting siRNA (Figure 5D). When condition media from Ang II-challenged cells was applied to control HUVECs, EndMT switch was evident (Figure 5E, Supplementary Figure 6). However, TLR2 deficiency was not able to offer protection against Ang II condition media, indicating that inflammatory and other paracrine factors released from cells activate EndMT through a TLR2-independent mechanism. Taken together, our results suggest that TLR2 signaling regulates Ang II-induced NF-κB activation and inflammatory cytokine release, and subsequently mediates EndMT. However, TLR2 may not be able to circumvent EndMT switch triggered by inflammatory factors and other mechanisms.

## DISCUSSION

**Figure 6:**
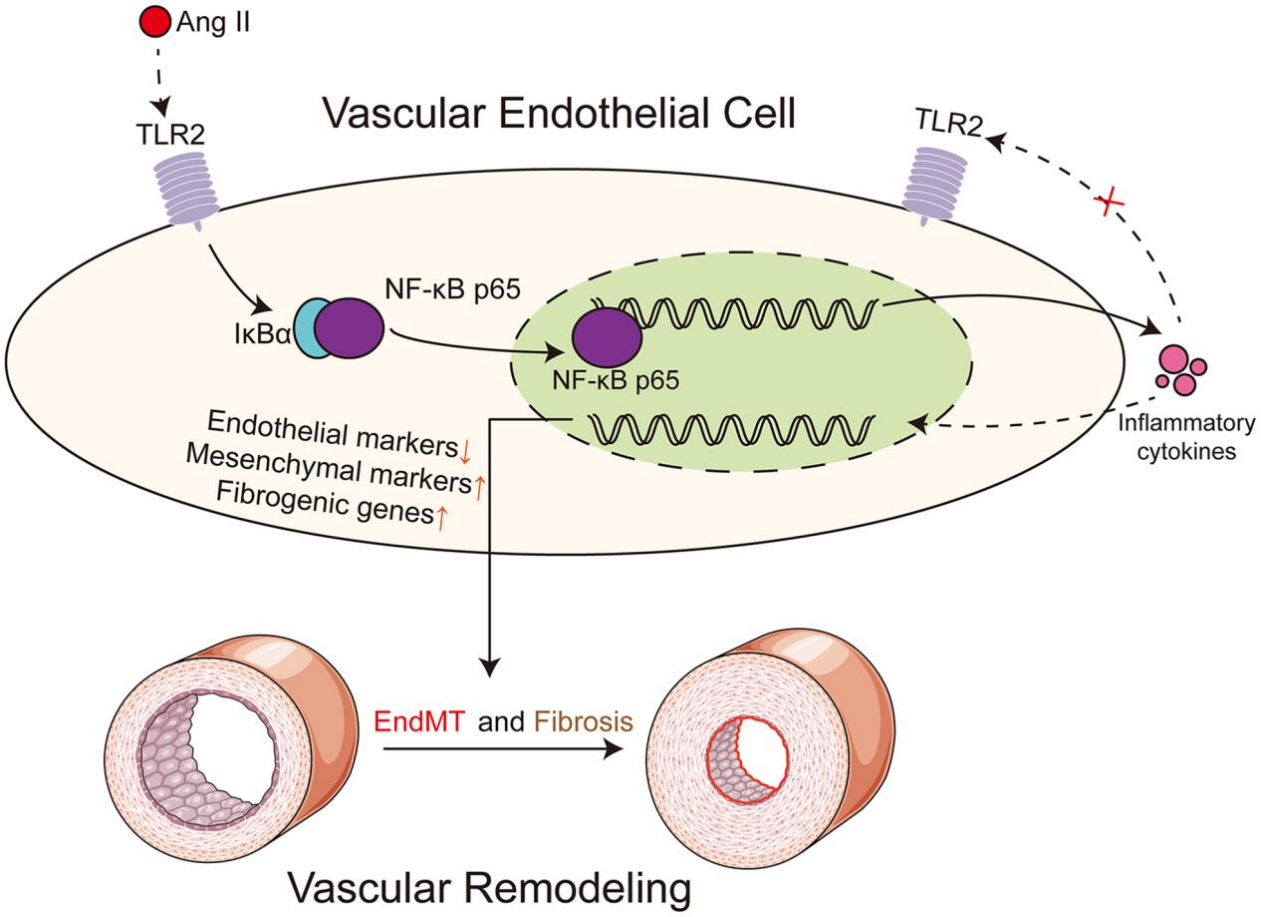
**Schematic illustration of the main findings.** Ang II potentially engages TLR2 signaling pathway to activate NF-κB in vascular endothelial cells. NF-κB orchestrates the induction of EndMT transcription factors to reduce endothelial phenotype makeup and increase mesenchymal genes. In addition to regulating cell phenotype change, NF-κB increases the expression of extracellular matrix proteins. Ang II may cause the production and release of paracrine factors which may further facilitate EndMT in a TLR2-independent manner. Dotted lines indicate putative mechanisms where direct evidence of the underlying phenomenon is needed. These putative mechanisms include evidence that Ang II directly engages TLR2 and receptors or sensors involved in detecting and responding to Ang II-generated paracrine factors.

In our study, we have shown that deficiency of TLR2 suppresses the activation of NF-κB in response to Ang II administration. This signaling pathway is critically involved in the secretion of inflammatory cytokines, deposition of matrix proteins, and initiation of EndMT. Deficiency in TLR2 largely prevents the development of these pathogenic changes in aortas. A schematic illustrating the main findings is shown in Figure 6.

Elevated Ang II has been linked to heart damage and kidney injury, as well as to vascular injuries [22–24]. Recent evidence has also shown that TLR2 regulates Ang II-induced cellular and tissue damage [14, 15, 24, 25]. TLR2 deficiency or inhibition is able to improve Ang II-induced cardiac damage, in part by suppressing the infiltration of macrophages [15]. Yan and colleagues also found that blocking TLR2 signaling decreases Ang II-induced chronic inflammation and vascular remodeling in models of abdominal aortic aneurysm [25]. Furthermore, miR-144-5p was found to regulate TLR2 signaling and the progression of aortic aneurysms following Ang II administration in apolipoprotein E knockout mice [24]. These studies suggest that some of the adverse effects of Ang II in vascular tissues may be regulated by TLR2. In agreement with this role, we show that mice deficient in TLR2 do not exhibit aortic lesions that are seen by chronic Ang II administration.

One mechanism that we have not been able to elucidate is how Ang II engages TLR2. It is unknown whether there is a direct interaction between Ang II and TLR2 or indirect activation by elaboration of TLR2 ligands, which may be generated through Ang II signaling. Based on our current understanding, it is possible that both hemodynamic and nonhemodynamic actions of Ang II are involved in TLR2 activation and vascular remodeling. For example, increased shear stress may be involved [26]. Inflammatory responses and increased oxidative stress may also contribute to vascular remodeling, independent of the regulation of blood pressure [22]. In support of this mechanism, Steven and colleagues showed that caveolin-1 deletion prevents vascular inflammation and remodeling, independent of systolic blood pressure changes in mice [27]. Ang II may potentially increase oxidative stress through Ang II receptors in vascular cells [28, 29]. In our study, we report that TLR2 knockout mice do not show any alteration in Ang II-induced systolic blood pressure, which was comparable to wildtype mice and consistent with previous reports [15]. It remains to be determined whether preventing inflammatory responses, increased oxidative stress, or Ang II receptors is able to prevent TLR2 activation.

The developmental EndMT process is gaining traction as being intimately involved in various forms of vascular injury [18, 30, 31]. Cardiovascular conditions such as cardiac fibrosis and atherosclerosis show induction of EndMT [32]. Furthermore, Ang II has been found to induce EndMT, in part through TGF-β signaling [33]. Inflammatory factors may also mediate Ang II-induced EndMT [34]. We empirically showed the association of TGF-β1, TGF-β1 signaling proteins Smad2/3, as well as induction of inflammatory cytokines by Ang II in endothelial cells and in aortas of mice. Removing TLR2 from the equation prevents the alteration of these various signaling axes. Furthermore, the downstream manifestation of these signaling aberrations, EndMT, is prevented when TLR2 is not present. It would be interesting to examine whether blocking signaling pathways singularly, for example inflammatory factors alone or TGF-β1 alone, would alter Ang II-induced EndMT or whether this complex cellular phenotype switch requires convergence of all activating axes. At least one aspect is clear in our study: the requirement of NF-κB to bring about these changes. NF-κB may be activated by TGF-β1, oxidative stress, inflammatory factors, Ang II receptor signaling, as well as by TLRs. Every pathway we have examined is easily linked to NF-κB. Other researchers have also shown that NF-κB activation increases the levels of α-SMA [35]. Li et al. demonstrated that activation of NF-κB regulates EndMT in type 1 diabetes [36]. Furthermore, Maleszewska et al. showed that TGF-β2 and IL-1β induce EndMT in a NF-κB-dependent manner [37]. Our study can be added to this list in implicating NF-κB as a central hub for EndMT initiation.

Increased inflammatory cytokines and other paracrine factors released in response to Ang II may also cause EndMT. Early studies conducted by Montesano and colleagues in the 1980s showed that condition medium from activated lymphocytes induces a fibroblast-like morphology in HUVECs [38]. Exposure of endothelial cells to TNF-α and IL-1β also induces EndMT [39, 40]. Moreover, Sánchez et al. found that exposure of endothelial cells to TNF-α or IL-1β individually is sufficient to change the cell phenotype in human primary aortic endothelial cells [30]. Although we noted increased mRNA levels of *Tnfa*, *Il6*, and *Il1b*, we did not test whether neutralizing these factors would reduce Ang II-induced EndMT. Furthermore, our studies indicate that TLR2 deficiency is unable to prevent EndMT induced by condition media from cells that had been challenged with Ang II. The likely explanation is that TLR2 deficiency fails to inhibit NF-κB, which is activated by inflammatory and other paracrine factors in the condition media. Other possibilities include autophagy [41], and activation of protein kinase B (Akt) and mitogen-activated protein kinase pathways [42], which may be at play. A comprehensive understanding of these inducers will greatly aid the identification of interventional and effective therapies.

A limitation of our studies that needs a mention is that we are unable to examine mRNA and proteins exclusively from endothelial cells in aortic tissues. Our *in vivo* studies utilized whole tissue lysates that would comprise vascular smooth muscle cells and infiltrating inflammatory cells. Therefore, based on *in vivo* studies alone, we are unable to conclude that Ang II induces inflammatory factors in endothelial cells. To circumvent this limitation, we carried out immunofluorescence staining of tissues to focus on the endothelial lining of aortas. In addition, we utilized the purified endothelial culture system to offer such insights. In particular, we show that Ang II induces EndMT in purified endothelial cells and that TLR2 knockdown negates these changes. We also should that Ang II induces inflammatory factors in cultured endothelial cells. When considered together with the *in vivo* studies, our hypothesis appears to hold true. Future studies utilizing endothelial-specific TLR2 knockout would be extremely beneficial in advancing this field.

In summary, we found that Ang II causes EndMT in aortas of mice and cultured cells through activating TLR2. We also report that NF-κB may mediate most of the pathological changes seen such as increased extracellular matrix deposition and elaboration of inflammatory cytokines. Deficiency in TLR2 downregulates the activation of NF-κB, thereby reliving the injury, possibly through suppression of EndMT. These studies suggest that TLR2 and NF-κB may be promising in preventing hypertension-associated vascular diseases.

## MATERIALS AND METHODS

### Reagents

Human umbilical vein endothelial cells (HUVECs) were obtained from the Shanghai Institute of Biochemistry and Cell Biology (Shanghai, China). HUVECs were cultured in Dulbecco’s modified Eagle 110 medium with 10% fetal bovine serum, 100 U/mL penicillin and 100 U/mL streptomycin at 37° C in a humidified 5 % CO_2_ incubator.

Ang II (>90% purity) was purchased from Aladdin (Shanghai China). NF-κB inhibitor (E)-3-(4-t-Butylphenylsulfonyl)-2-propenenitrile (Bay 11-7085; B5681) was obtained from Sigma (St. Louis, MO, USA). Antibodies against inhibitor of κB-α (IκB; 4812S), NF-κB p65 (8242S), TLR2 (12276) and GADPH (5174) were purchased from Cell Signaling Technology (Boston, USA). Antibodies against phosphorylated-Smad2/3 (p-Smad2/3; sc-11769) were obtained from Santa Cruz Biotechnology (CA, USA). Lamin B (ab133741), tumor necrosis factor-α (TNF-α; (ab6671), transforming growth factor-β1 (TGF-β1; ab92486), Col-3 (ab7778), Vimentin (ab8978), Snail/Slug (ab180714), smooth muscle actin (α-SMA; ab5694), vascular endothelial cadherin (VE-cadherin; ab9104), Twist (ab50581) and CD31 (ab28364, ab6543) were obtained from Abcam (Cambridge, UK). Secondary antibodies were obtained from Yeasen Biotech (Shanghai, China).

### Ang II-induced vascular remodeling in mice

All animal care and experimental procedures were approved by the Wenzhou Medical University Animal Policy and Welfare Committee (Approval Document No. wydw2016-0124). All animals received humane care according to the National Institutes of Health (USA) guidelines. Studies followed the ARRIVE guidelines for reporting experiments involving animals [43].

C57BL/6 male mice were obtained from Animal Center of Wenzhou Medical University. Male TLR2 knockout (TLR2 KO; B6.129-TLR2tm1Kir/J) on C57BL/6 background were obtained from the Jackson Laboratory (Bar Harbor, ME, USA). Eight-week-old mice (15 C57BL/6 mice and 13 TLR2KO mice) were housed with a 12:12 h light–dark cycle at a constant room temperature and fed a standard rodent diet. The animals were acclimatized to the laboratory for at least 2 weeks before initiating the studies.

WT and TLR2 KO mice were divided into 2 groups each: untreated C57BL/6 mice (*WT*, n = 8), untreated TLR2 KO mice (n = 6), WT mice treated with Ang II (n = 7), and TLR2 KO mice treated with Ang II (n = 7). Ang II was administered using a micro-osmotic pump (1002, Alzet, USA) as previously reported [44]. The pump was implanted subcutaneously in the back of mice and delivered 1000 ng/kg/min Ang II for 2 weeks. Systolic blood pressure was measured weekly by tail-cuff using the telemetric blood pressure system (BP-2010A, Softron Biotechnology, Tokyo, Japan), as described previously [15]. Measurements were performed during daytime (1:00 pm to 5:00 pm) with previous 5 days of training. At the conclusion of the study, mice were sacrificed, and aortas were excised. Serum levels of interleukin-6 (IL-6) were detected by ELISA (85-88-7064-76; Thermo Fisher).

### Histology and tissue staining

Tissues were embedded in optical cutting temperature compound (Sakura, Japan). Five micron-thick sections were stained with hematoxylin and eosin (H&E) to assess intimal thickening. Sections were also stained with Masson's Trichrome staining to examine fibrosis. For immunohistochemical staining, tissue sections fixed. Endogenous peroxidase activity was quenched with 0.3% hydrogen peroxide. Slides were blocked in 5% bovine serum albumin for 30 min. Primary antibodies (collagen III, 1:500; TNF-α, 1:500) were then applied at 4° C overnight. Horseradish peroxidase-conjugated secondary antibodies and diaminobenzidine (DAB) was used for detecting immunoreactivity. Slides were counterstained with hematoxylin.

For immunofluorescence staining, 5-μm tissue sections were used. Cells cultured on gelatin-coated glass slides were also stained. Slides were permeabilized with 0.1% Triton X-100 for 10 minutes and blocked with 5% bovine serum albumin for 45 minutes. Primary antibodies were applied at 4° C overnight. These antibodies included CD31 (1:200), vimentin (1:200), α-SMA (1:200), VE-Cadherin (1:200), and NF-κB p65 (1:200). Alexa Fluor-488, Alexa Fluor-TRITC, and Alexa Fluor-647 were used for detection. Slides were counterstained with DAPI for 5 min. For double labeling, antibodies (primary and secondary) were applied sequentially. Fluorescence images were acquired using Leica TCS SP8 confocal laser scanning microscope (Buffalo Grove, USA). Nikon microscope (Nikon, Japan) was used for brightfield images.

### TLR2 silencing

TLR2 was silenced in HUVECs by siRNA transfection. TLR2 siRNA sequences were purchased from Gene Pharma Co. LTD. (Shanghai, China). Sequence is provided in Supplementary Table 1. Control transfections were performed with scrambled siRNA sequences (Gene Pharma). siRNAs were transfected using LipofectAMINE 2000 (Thermo Fisher, Carlsbad, CA).

### Generation of NF-κB EGFP expressing cells

Stable NF-κB reporter transfections were performed in HUVECs using pGL3-NF-κB-EGFP lentiviral particles. Lentivirus containing the response element (RE) of NF-κB was first obtained by transfecting HEK-293T cells with p-LV-NFκB-RE-EGFP (Thermo Fisher) using Polyethylenimine (Sigma). Supernatant was collected 48 h later and passed through a 0.45 μm filter. Then, HUVECs were incubated with the supernatant and 8 μg/mL polybrene (Sigma-Aldrich) for 12 h. To select for transfected cells, 1 μg/mL puromycin (Thermo Fisher) was added to the culture media. Following purification of NF-κB reporter expressing cells, cells were transfected with scrambled siRNA or TLR2 siRNA. Cells were then exposed to 10 μM Ang II for 2 h. The EGFP signal was determined from florescence images.

### Real-time quantitative PCR

Total RNA was isolated from cultured cells and aortic vessels using TRIZOL (Thermo Fisher). Reverse transcription and quantitative PCR was carried out using two-step PrimeScript RT reagent Kit (Perfect Real Time; TAKARA) and Eppendorf Mastercycler ep realplex detection system (Eppendorf, Hamburg, Germany). Primers for genes were synthesized and obtained from Thermo Fisher. Sequences are presented in Supplementary Table 1. mRNA of target genes was normalized to β-actin housekeeping gene.

### Western blot analysis

Sixty micrograms of cell and tissue lysates were separated by 10% SDS-PAGE and electro-transferred to a PVDF membranes. Membranes were blocked in Tris-buffered saline containing 0.05% Tween20 and 5% non-fat milk for 1.5 h. PVDF membranes were then incubated with specific primary antibodies. Immunoreactive bands were detected by incubating with secondary antibodies conjugated to horseradish peroxidase and enhanced chemi-luminescence reagent (Bio-Rad). For some studies, cytosolic and nuclear fractions were separated by using nuclear protein extraction kit (Beyotime Biotech). For these studies, a total of 20 μg nuclear proteins and 40 μg cytosolic proteins were used for immunoblotting. Densitometric quantification was performed using Image J analysis software version 1.38e and normalized to their respective control (GAPDH for cytosolic proteins and Lamin B for nuclear fractions).

### Statistical analysis

Data shown is from 3 independent experiments, each with 3 replicates for cell studies and 6-8 replicates for mouse studies. Data is expressed as Mean ± SEM. Statistical analysis was performed with GraphPad Prism 8.0 software (San Diego, CA, USA). We used one-way ANOVA followed by Dunnett's post hoc test when comparing more than two groups of data. P values of < 0.05 were considered statistically significant. Post-tests were run if F achieved *p* < 0.05 and there was no significant variance in homogeneity.

## Supplementary Material

Supplementary Figures

Supplementary Table 1
