# Supplementary material for: TLR2 regulates angiotensin II-induced vascular remodeling and EndMT through NF-κB signaling
Source: Aging (Albany NY). 2020 Dec 9;13(2):2553–74. doi: 10.18632/aging.202290 (PMC7880316; doi:10.18632/aging.202290)
Supplement: Supplementary Figures [file aging-13-202290-s001.pdf]

SUPPLEMENTARY FIGURES

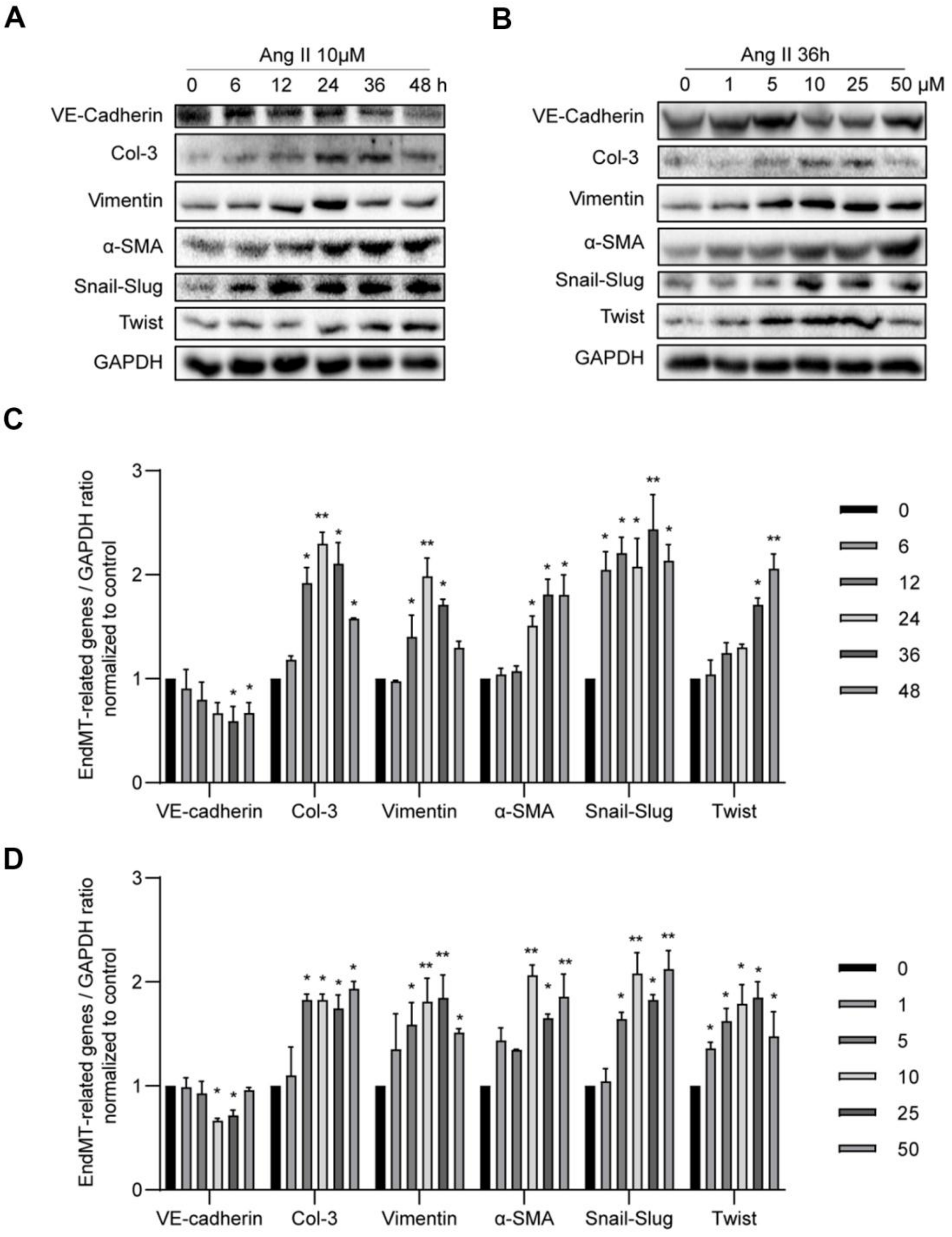

Supplementary Figure 1. Ang II induces EndMT in cultured endothelial cells. (**A**) HUVECs were exposed to 10 μM Ang II for various time points. PBS was used as vehicle control. Cell lysates were subjected to immunoblotting to assess EndMT. Levels of endothelial marker VE-cadherin, mesenchymal markers α-SMA and Vimentin, EndMT-associated transcription factors Snail/Slug and Twist, and extracellular protein Collagen-3 were detected. GAPDH was used as loading control. (**B**) HUVECs were exposed to varying concentrations of Ang II for 36 h. Lysates were used for western blot as described in panel A. (**C**) Densitometric quantification of blots shown in panel A. (**D**) Densitometric quantification of EndMT-associated marker shown in panel B. [n = 3 independent experiments; *p<0.05 and **p<0.01 compared to control (0)].

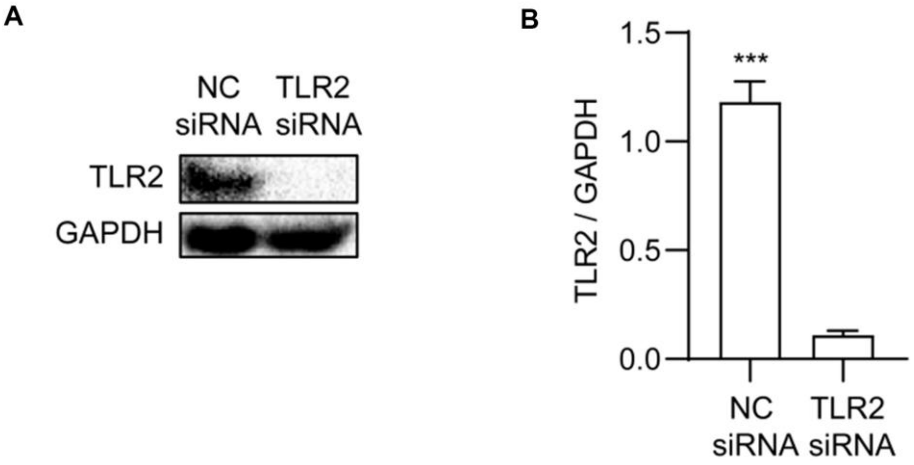

Supplementary Figure 2. TLR2 silencing in endothelial cells. HUVECs were transfected with negative control (NC) siRNA or siRNA against TLR2. Total proteins were probed for levels of TLR2. GAPDH was used as loading control. (**A**) representative immunoblot of TLR2. (**B**) Densitometric quantification of TLR2 protein levels following siRNA transfection. [n = 3; Data shown as Mean ± SEM; ***p<0.001 compared to control transfection].

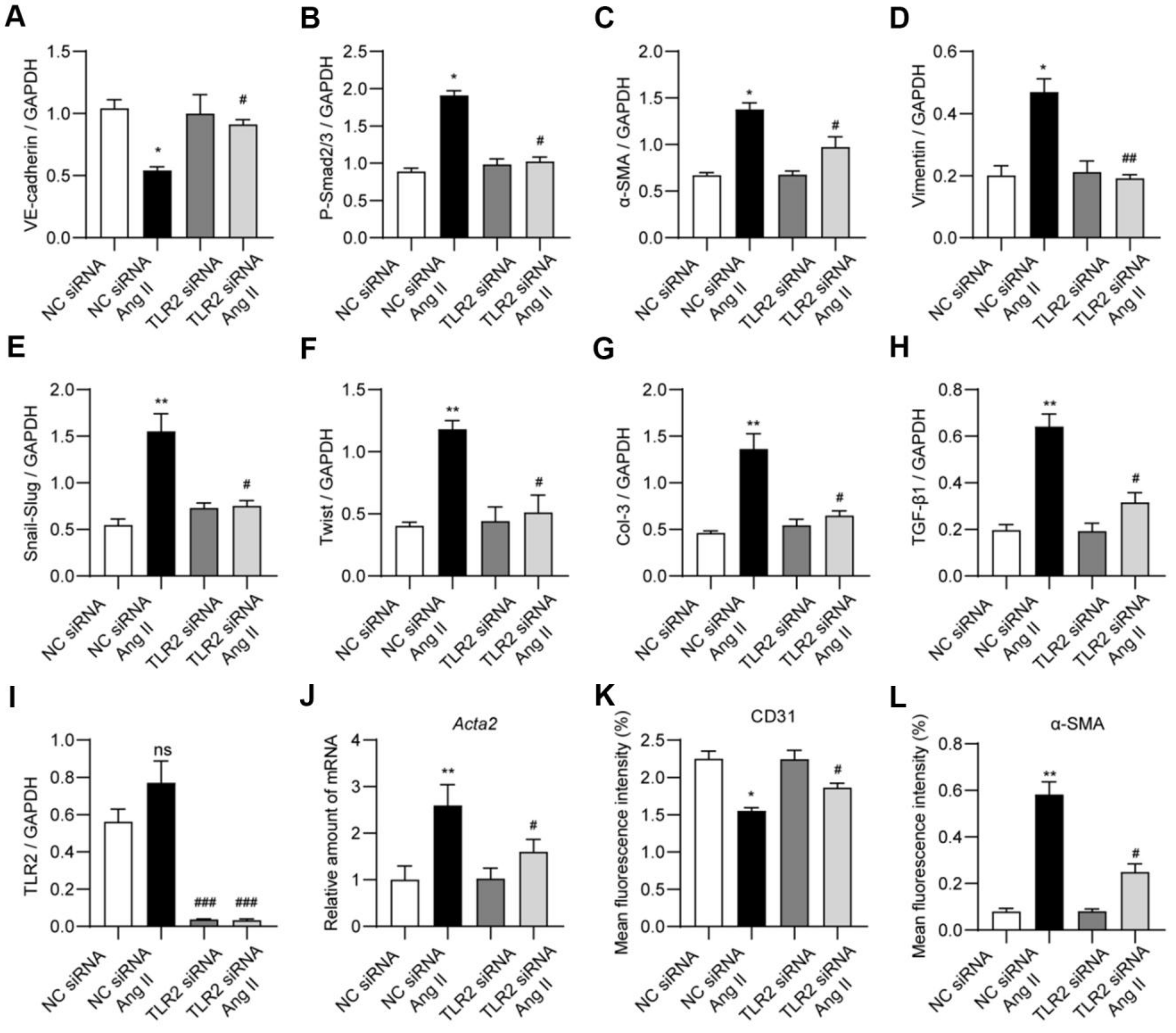

Supplementary Figure 3. TLR2 silencing prevents Ang II-induced EndMT protein induction. (**A**–**I**) Densitometric quantification of EndMT-associated proteins in HUVECs following TLR2 silencing. HUVECs were transfected with NC siRNA or TLR2 siRNA and then exposed to 10 µM Ang II for 36 h. Data was normalized to GAPDH. Representative western blots are presented in Figure 3A. (**J**) mRNA levels of *Acta2* (α-SMA) in HUVECs exposed to 10 µM Ang II for 24 h following TLR2 siRNA transfection. (**K**, **L**) Quantification of CD31 (**K**) and α-SMA (**L**) staining of HUVECs. Representative fluorescence images are shown in Figure 3D. [n = 3; Data shown as Mean ± SEM; *p<0.05 and **p<0.01 compared to negative control siRNA transfection; #p<0.05, ##p<0.01, and ###p<0.001 compared to NC siRNA + Ang II].

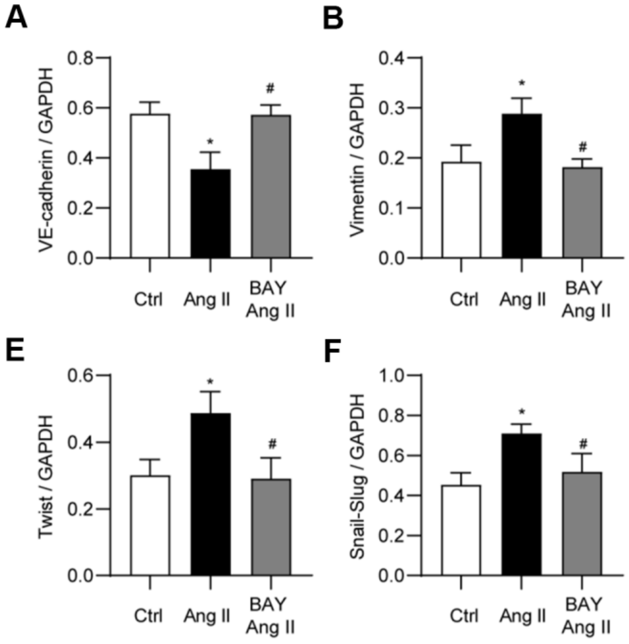

Supplementary Figure 4. NF-κB inhibition prevents Ang II-induced EndMT in cultured cells. (**A**–**F**) Densitometric quantification of EndMT-associated proteins in HUVECs treated with NFκB inhibitor Bay 11-7085 at 5 μM, before exposure to 10 μM Ang II for 36 h. Representative immunoblots are shown in Figure 4A. Data normalized to GAPDH. [n = 3; Data shown as Mean ± SEM; *p<0.05 compared to Control; #p<0.05 and ##p<0.01 compared to Ang II].

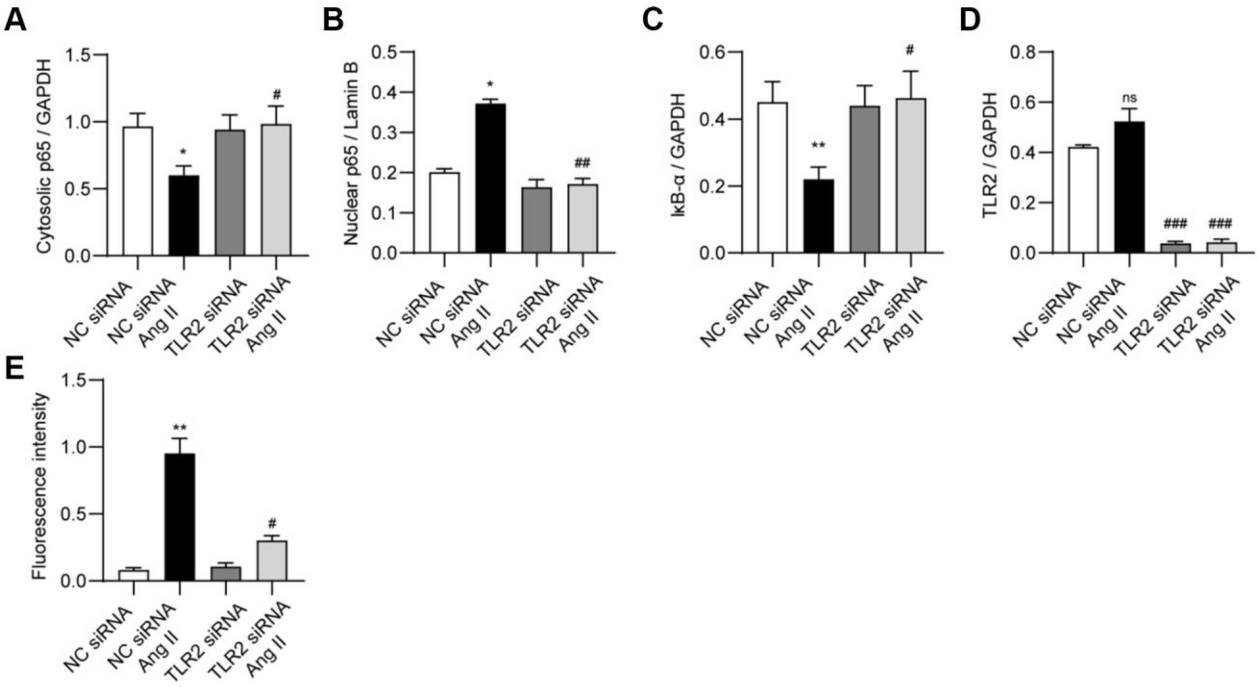

Supplementary Figure 5. TLR2 regulates Ang II-induced NF-κB signaling. (**A**–**C**) Densitometric quantification of NF-κB signaling proteins in HUVECs transfected with TLR2 siRNA and exposed to 10 μM Ang II for 2 h. Data showing levels of cytosolic p65 subunit of NF-κB (**A**), nuclear p65 levels (**B**), and inhibitor of κB in total lysates (**C**). Data normalized to Lamin B for nuclear proteins and GAPDH for cytosolic proteins. Representative immunoblots are shown in Figure 4B. (**D**) Proteins levels of TLR2 in HUVECs transfected with TLR2 siRNA and exposed to 10 μM Ang II for 2 h. Data normalized to GAPDH. Representative immunoblots are shown in Figure 4B. (**E**) Quantitation of EGFP fluorescence intensity in HUVECs transfected with NF-κB reporter. Representative EGFP images are shown in Figure 4E. [n = 3; Data shown as Mean ± SEM; *p<0.05 and **p<0.01 compared to control NC siRNA; #p<0.05, ##p<0.01, and ###p<0.001 compared to NC siRNA+Ang II].

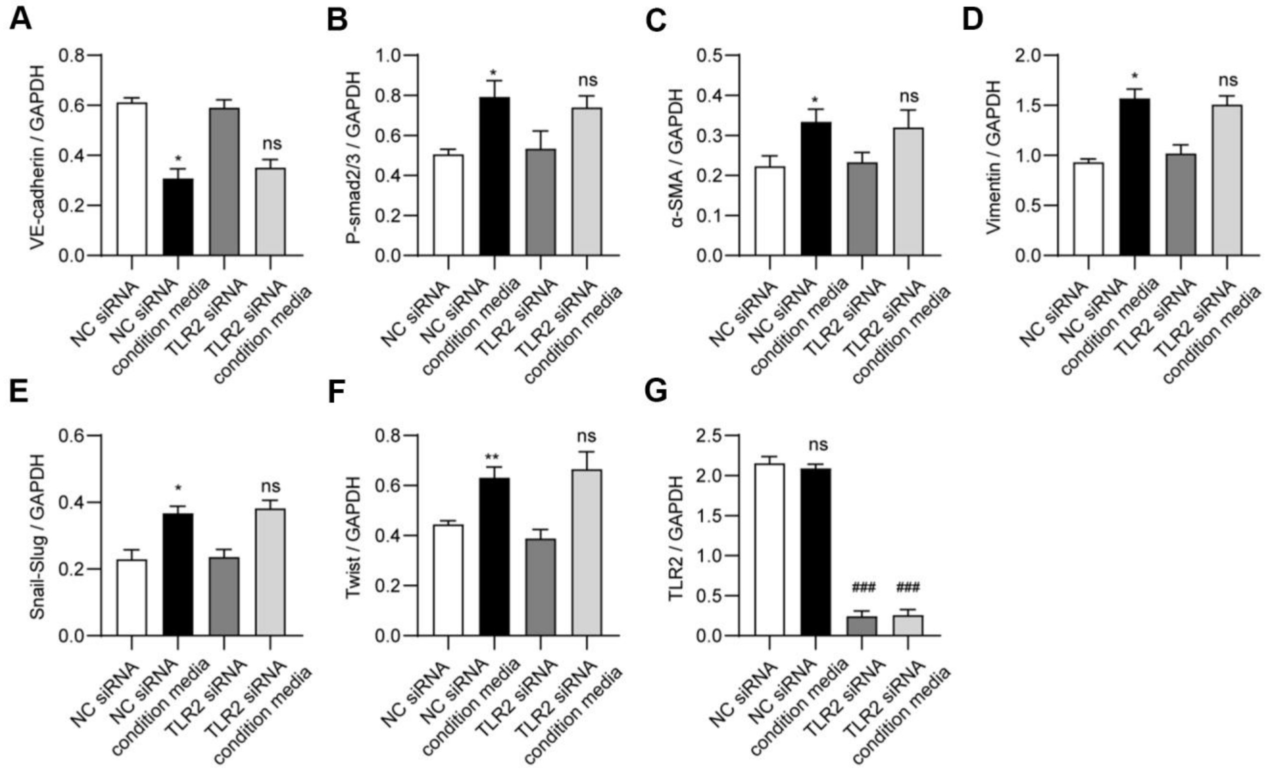

Supplementary Figure 6. TLR2 deficiency does not prevent EndMT induced by paracrine factors generated upon Ang II challenge. (**A**–**G**) Densitometric quantification of EndMT-associated proteins in HUVECs exposed to condition media from Ang II-challenged cells. Experimental setup is shown in Figure 5D and representative blots are shown in Figure 5E. Data normalized to GAPDH. [n = 3; Data shown as Mean ± SEM; *p<0.05 and **p<0.01 compared to Control media and NC siRNA; ###p<0.001 compared to NC siRNA; ns = not significant, compared to NC siRNA condition media].
