# Supplementary material for: TLR2 regulates angiotensin II-induced vascular remodeling and EndMT through NF-κB signaling
Source: Aging (Albany NY). 2020 Dec 9;13(2):2553–74. doi: 10.18632/aging.202290 (PMC7880316; doi:10.18632/aging.202290)
Supplement: Supplementary Table 1 [file aging-13-202290-s002.pdf]

SUPPLEMENTARY TABLE

Supplementary Table 1. Nucleotide sequences for qPCR and gene silencing.

Gene	Species	Description	Sequence
Col3a1	Human	Collagen type III alpha 1 chain	GGAGCTGGCTACTTCTCGC<br>GGGAACATCCTCCTTCAACAG
Tgfb1	Human	Transforming growth factor β1	GGCCAGATCCTGTCCAAGC<br>GTGGGTTTCCACCATTAGCAC
Vim	Human	Vimentin	TCCAGCAGCTTCCTGTAGGT<br>CCCTCACCTGTGAAGTGGAT
Cdh5	Human	Vascular endothelial cadherin	CGAGAGCTACACGTTCACGG<br>GGGTGTCGAGGGAAAAATAGG
Acta2	Human	Smooth muscle actin (α-SMA)	AGAGTTACGAGTTGCCTGATG<br>ATGAAGGATGGCTGGAACAG
Tnfa	Human	Tumor necrosis factor α	CCTCTCTCTAATCAGCCCTCTG<br>GAGGACCTGGGAGTAGATGAG
Il1b	Human	Interleukin 1β	ACGCTCCGGGACTCACAGCA<br>TGAGGCCCAAGGCCACAGGT
Il6	Human	Interleukin 6	AACCTGAACCTTCCAAAGATGG<br>TCTGGCTTGTTCCTCACTACT
Actb	Human	β actin	CCTGGCACCCAGCACAAT<br>GCCGATCCACACGGAGTACT
Col3a1	Mouse	Collagen type III alpha 1 chain	CTGTAACATGGAAACTGGGGAAA<br>CCATAGCTGAACTGAAAACCACC
Tgfb1	Mouse	Transforming growth factor β1	TGACGTCACTGGAGTTGTACGG<br>GGTTCATGTCATGGATGGTGC
Vim	Mouse	Vimentin	CGTCCACACGCACCTACAG<br>GGGGGATGAGGAATAGAGGCT
Snai1	Mouse	Snail family transcriptional repressor 1	CACACGCTGCCTTGTGTCT<br>GGTCAGCAAAAGCACGGTT
Snai2	Mouse	Snail family transcriptional repressor 2 (Slug)	CATCCTTGGGGCGTGTAAGTC<br>GCCCAGAGAACGTAGAATAGGTC
Cdh5	Mouse	Vascular endothelial cadherin	CACTGCTTTGGGAGCCTTC<br>GGGGCAGCGATTCATTTTTCT
Twist1	Mouse	Twist family BHLH transcription factor 1	GGACAAGCTGAGCAAGATTCA<br>CGGAGAAGGCGTAGCTGAG
Il1b	Mouse	Interleukin 1β	ACTCCTTAGTCCTCGGCCA<br>CCATCAGAGGCAAGGAGGAA
Il6	Mouse	Interleukin 6	GAGGATACCACTCCCAACAGACC<br>AAGTGCATCATCGTTGTTCATACA
Tnfa	Mouse	Tumor necrosis factor α	CAGGGGCCACCACGCTCTTC<br>TTTGTGAGTGTGAGGGTCTGG
Actb	Mouse	β actin	CCGTGAAAAGATGACCCAGA<br>TACGACCAGAGGCATACAG
TLR2	Human	Toll like receptor 2 (siRNA sequence)	GCCCUCUCUACAAACUUUATT<br>UAAAGUUUGUAGAGAGGGCTT
NC	Human	Negative control (siRNA sequence)	UUCUCCGAACGUGUCACGUTT<br>ACGUGACACGUUCGGAGAATT
